# Molecular Mechanisms and Clinical Manifestations of Catecholamine Dysfunction in the Eye in Parkinson’s Disease As a Basis for Developing Early Diagnosis

**DOI:** 10.32607/actanaturae.10906

**Published:** 2020

**Authors:** T. A. Pavlenko, N. B. Chesnokova, M. R. Nodel, A. R. Kim, M. V. Ugrumov

**Affiliations:** Helmholtz Moscow Research Institute of Eye Diseases of Ministry of Health of the Russian Federation, Moscow, 105062 Russia; Sechenov First Moscow State Medical University, Moscow, 119991 Russia; Pirogov Russian National Research Medical University, Russian Clinical and Research Center of Gerontology, Moscow, 129226 Russia; Koltzov Institute of Developmental Biology of Russian Academy of Sciences, Moscow, 119334 Russia

**Keywords:** Parkinson’s disease, dopamine, lacrimal fluid, intraocular pressure, biomarkers

## Abstract

This review provides information on the non-motor peripheral manifestations of Parkinson’s disease (PD) associated with a pathology of the visual analyzer and the auxiliary apparatus of the eye. The relationship between neurodegenerative processes that take place in the brain and in the eye opens new prospects to use preventive ophthalmologic examination to diagnose PD long before the characteristic motor symptoms appear. This will encourage the use of neuroprotective therapy, which stops, or at least slows down, neuronal death, instead of the current replacement therapy with dopamine agonists. An important result of an eye examination of patients with PD may be a non-invasive identification of new peripheral biomarkers manifesting themselves as changes in the composition of the lacrimal fluid.

## 1. INTRODUCTION

The number of people suffering from neurodegenerative diseases continues to grow, while treatment and care for such patients remain a vexing social and medical problem. According to the WHO, by 2030 the number of people with neurodegenerative diseases in the world will rise to 75.5 million and exceed 135 million by 2050 [1-4].

Parkinson’s disease (PD) is a chronic progressive neurodegenerative disease that is clinically diagnosed mainly through a characteristic impairment of the motor function, concurrently with a systemic pathological process [4]. Therefore, one of the main priorities in neurology is to develop an early diagnosis of PD (before mobility impairments appears) that is based on a search for non-motor symptoms and markers in body fluids.

The pathogenetic mechanisms responsible for the development of motor symptoms are based on a progressive death of the dopaminergic neurons in the substantia nigra pars compacta, one of the key components of motor function regulation. The disease develops asymptomatically for years or even decades (up to the age of 30), while the mobility impairments that allow one to diagnose PD manifest themselves only after more than half of the dopaminergic neurons of the substantia nigra have died [5].

The key role in the pathogenesis of both the sporadic (polygenic) and hereditary (monogenic) forms of PD is played by an internal neuronal accumulation of the α-synuclein protein, which turns toxic upon aggregation. This leads to mitochondrial dysfunction, oxidative stress, and neuronal death. Neurodegeneration is triggered, or at least potentiated, by neuroinflammation, which is based on microglia activation. This increases the secretion of pro-inflammatory factors, leading to the death of both dopaminergic and other neurons (serotonin, norepinephrine, acetylcholinergic, and other neurons) [6-10]. In patients with PD, neurodegeneration is systemic and involves not only the nigrostriatal system, but also the monoaminergic nuclear structures of the brainstem, the limbic system, the cerebral cortex, as well as the structures of the peripheral (mainly sympathetic) nervous system, which underlies a wide range of non-motor disorders [11]. As a result, both the brain and the peripheral organs can be a source of biomarkers at the early stage of PD [7, 8, 9]. In this regard, the eye is of particular interest, since dopaminergic, adrenergic, and noradrenergic elements are widely represented in the retina, the optic nerve (which shares a common embryonic origin with the central nervous system (CNS)), and the nigrostriatal system [10].

## 2. ORGANIZATION AND CATECHOLAMINERGIC REGULATION OF THE EYE IN ITS NORMAL STATE

The organ of sight consists of the eyeball, connected by the optic nerve to the brain, and the auxiliary apparatus (the eyelids, the lacrimal organs, and the extraocular muscles). Eye function is maintained by a complex neural regulation on different levels, from the auxiliary apparatus of the eye, pupil reaction to light, accommodation, photoreception, etc. to information processing in the higher visual center in the occipital lobes of the cerebral cortex [12-16].

**Fig. 1:**
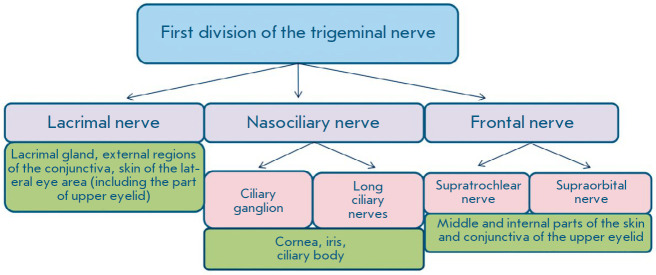
A diagram of the afferent innervation of the eye

Innervation of the eye is effected by both the parasympathetic and the sympathetic nervous systems. Sensitive innervation of the eye is triggered by the first division of the trigeminal nerve and the ophthalmic nerve, which enters the orbit through the superior orbital fissure and is divided into three branches: the lacrimal, the nasociliary, and the frontal nerves. The lacrimal nerve innervates the lacrimal gland, the external regions of the conjunctiva of the eyelids and eyeball, as well as the skin of the lower and upper eyelids. The nasociliary nerve is connected to the ciliary ganglion via a communicating branch; long ciliary nerves go to the eyeball. Finally, in the suprachoroidal space near the ciliary body, they form a dense plexus, with its branches penetrating the cornea. The frontal nerve is divided into two branches: the supraorbital and supratrochlear nerves. All branches, anastomose among themselves, thus innervate the middle and inner parts of the upper eyelid skin. The ciliary ganglion consists of sensitive fibers of the nasociliary nerve, parasympathetic fibers of the oculomotor nerve, and sympathetic fibers of the internal carotid plexus. The ciliary nerves arise from the ciliary ganglion and penetrate the eyeball through the posterior sclera. They supply the eye tissues with sensitive parasympathetic and sympathetic fibers. Parasympathetic fibers of the oculomotor nerve innervate the sphincter of the pupil and the ciliary muscle. The sympathetic fibers of the internal carotid plexus are connected to the iris dilator muscle (*Fig. 1*) [14, 17, 18].

**Fig. 2:**
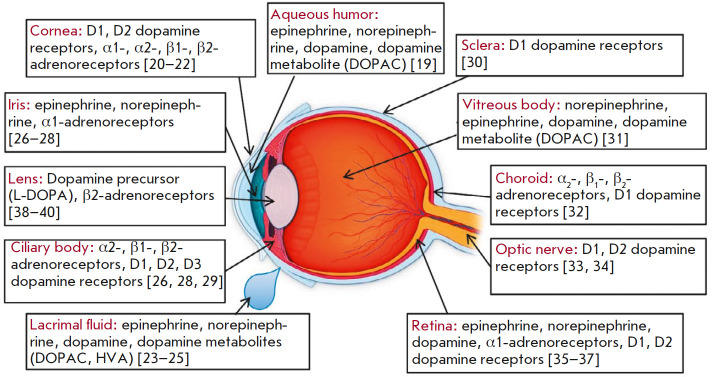
Catecholamines and receptors with which they interact in the eye

Catecholaminergic neurons, including the dopaminergic ones, were found in different parts of the visual system. In addition, catecholamines and their metabolites, as well as adrenoreceptors and dopamine receptors, were shown to be present in various eye structures (*Fig. 2*).

**Fig. 3:**
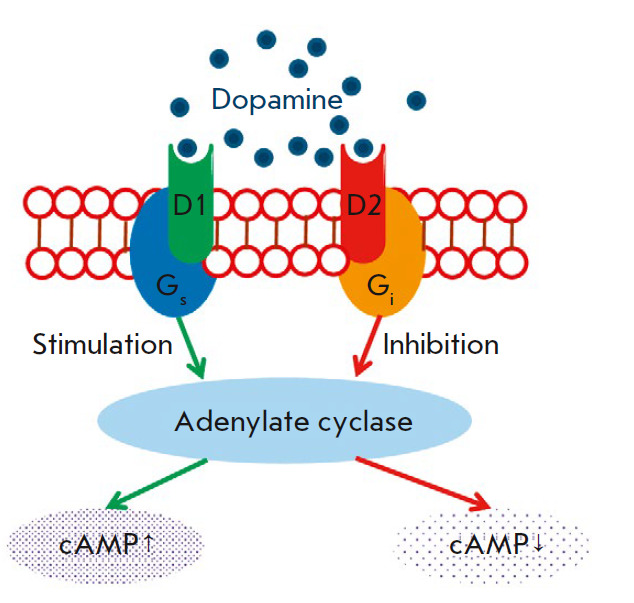
The mechanism of signal transmission from dopamine to the cell (D1 and D2 are dopamine receptors of the D1 and D2 families; cAMP stands for cyclic adenosine monophosphate)

In the eye, the action of DA on target cells is mediated by dopamine receptors: D1 receptors belonging to the D1 receptor family; as well as D2 and D3 receptors, from the D2 receptor family. It is known that receptors from the D1 family (D1 and D5) are conjugated to the Gs protein that stimulates adenylate cyclase, while receptors of the D2 family (D2, D3 and D4) are conjugated to the Gi protein that inhibits adenylate cyclase (*Fig. 3*). [41-44]. It has been experimentally shown that in high concentrations, DA also stimulates the α- and β-adrenergic receptors [45]. The same effect of DA on the adrenergic system can be achieved not only by direct stimulation of adrenergic receptors, but also through the ability of DA to stimulate the release of norepinephrine from granular presynaptic depots, which means that it can have an indirect adrenomimetic effect [43, 45].

**2.1. The retina**

**Fig. 4:**
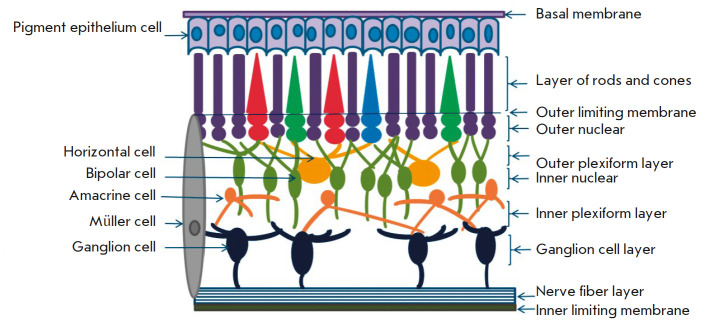
The retinal structure

The retina is a multilayer structure (*Fig. 4*) whose outer layer consists of photoreceptors (rods and cones, specialized highly differentiated cells) immersed into the pigment epithelium. Next comes the outer limiting membrane (a layer of intercellular adhesions), consisting of permeable, viscous, tightly fitting intertwined apical parts of the photoreceptors and Muller cells. The outer nuclear layer that comes next is formed by photoreceptor nuclei. It is followed by the outer plexiform layer located between the outer and inner nuclear layers. After that comes the inner nuclear layer, formed by the nuclei of bipolar, amacrine, horizontal, Muller, and interplexiform neurons. The inner plexiform layer, which consists of interwoven neuronal processes, separates the inner nuclear layer from the layer of ganglion cells. It delimits the vascular interior of the retina from the avascular exterior. Next is the layer formed by  retinal ganglion cells. The layer after that consists of ganglion cell axons forming the optic nerve. From the inside, the retinal surface is covered by the inner limiting membrane, in which the processes of neuroglial Muller cells take place [14, 18, 46].

Endogenous transmitters play an important role in the transmission of nervous excitation along retinal neurons. Almost all known neurotransmitters, including DA, are found in the retina. In the retina, DA is involved in visual perception, regulation of the circadian rhythm, and autoregulation of the vascular tone. Dopamine receptors are found in almost all retinal cells [47, 48]. In addition, DA is synthesized in many retinal cells: photoreceptors, amacrine, bipolar, interplexiform, and horizontal cells [32, 35, 36, 37, 49]. The highest DA concentration was found in amacrine cells, which are involved in horizontal momentum transfer from bipolar cells to amacrine, and further to retinal ganglion cells [36, 50]. It is important to note that the DA concentration, including that in the retina, can be affected by the α-synuclein neuronal protein, which is found mainly in presynaptic terminals. It performs a number of functions, participating in the regulation of the vesicular transport and affecting the intracellular concentration of dopamine as it inhibits tyrosine hydroxylase, the rate-limiting enzyme of dopamine synthesis [2, 51, 52]. In the retina, α-synuclein is found in the axons of photoreceptors; it is also expressed in the bipolar and amacrine cells of the retina and is present in the presynaptic terminals of neurons in the external and internal plexiform layers [53, 54].

Experimental studies have highlighted the important functional role played by dopaminergic retinal cells. Stutz B. et al. [55] showed that retinal cells implanted into the striatum can compensate for DA deficiency. So, when transplanting murine Muller cells containing dopamine into the striatum of other mice with Parkinsonism, the striatal DA level was normalized and the motor function was recovered. After transplanting the retinal pigment epithelial cell culture into the striatum of mice with Parkinsonism, an increased production of neurotrophic factors (glial and brain ones) occurred in the transplanted cells, thus protecting DA-containing cells from damage. In addition, it was shown on the rotenone-induced model of Parkinsonism that retinal pigment epithelial cells can synthesize DA, making up for its deficiency [56].

**2.2. The anterior segment of the eyeball**

**Fig. 5:**
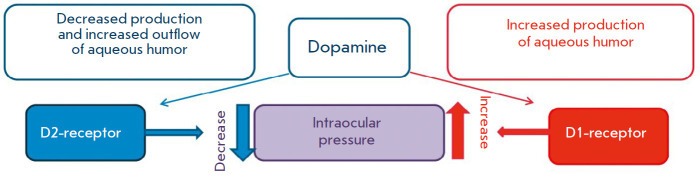
The effect of dopamine on intraocular pressure

The tissues of the anterior segment of the eye possess dopaminergic nerve fibers and DA receptors [29] (*Fig. 2*). According to various authors, the DA concentration in the intraocular fluid of the anterior chamber of the human eye ranges from 0.120 to 0.318 ng/ml [19, 57]. The dopaminergic system of the eye plays an important role in the regulation of intraocular pressure (IOP). Studies focused on the effects of DA and its agonists on IOP in humans and experimental animals led to a conclusion that this effect is complex, being direct at the postsynaptic level and indirect at the presynaptic level. The postsynaptic effect of DA agonists stimulates the α, β, and D1 receptors in the ciliary body, while the indirect effect is achieved with the help of the α2, D2, and D3 receptors. It was shown that D1 receptors are present in the ciliary body epithelium [58] and in the epithelium of the ciliary processes [44, 59, 60]; on the one hand, their stimulation increases the production of aqueous humor, while, on the other hand, it affects the tone of the ciliary muscle and changes the outflow of moisture. The D2 receptors are apparently mainly located in the postganglionic presynaptic nerve endings [61]. Experimental and clinical studies have shown that exposure to D1 receptor agonists increases the intraocular pressure, while D2 receptor agonists reduce IOP (*Fig. 5*) [19, 62, 63].

**2.3. Lacrimal organs, eyelids, and lacrimal fluid**

The lacrimal organs include the lacrimal gland, the accessory lacrimal glands, the lacrimal ducts, and the glands of the eyelid. All the secretions of the main and accessory lacrimal glands, as well as those of the glands of the eyelid, are involved in the formation of the lacrimal fluid and the lacrimal film covering the anterior corneal surface. The lacrimal fluid is produced in the main and accessory lacrimal glands. The lacrimal gland (a tubuloacinar exocrine gland) secretes electrolytes, water, proteins with various functions, and mucins as components of the lacrimal fluid and the lacrimal film.

Tear secretion can be basal or reflexive. Basal tears are secreted by many lacrimal glands. They are the accessory lacrimal glands of Krause or Wolfring, as well as the glands of the semilunar fold and the lacrimal caruncle. The meibomian glands and the glands of Zeis and Moll produce lipid secretum, while mucous glands secrete mucin (goblet cells, conjunctival epithelial cells, the crypts of Henle in the tarsal part of the conjunctiva, and the glands of Manz in the limbal conjunctiva) [14, 64, 65, 66]. Reflexive tear secretion is effected by the main lacrimal gland and regulated mainly by the trigeminal parasympathetic reflex resulting from psychogenic stimulation, exposure to bright light, or in response to irritation of the conjunctiva or cornea. The parasympathetic system is believed to prevail in the innervation of the lacrimal gland [14, 64, 65, 66]. The afferent innervation of the lacrimal gland is effectuated by the trigeminal nerve system:* n. lacrimalis *(a branch of *n. ophtalmicus*). The efferent parasympathetic innervation of the lacrimal gland is made possible by the branches of the trigeminal nerve and by the parasympathetic fibers in the facial nerve system.

The parasympathetic neurotransmitter acetylcholine and the sympathetic neurotransmitter norepinephrine are the main neurotransmitters that regulate tear secretion [64, 65, 66, 67]. The involvement of other neurotransmitters of the sympathetic system in the innervation of the lacrimal gland is confirmed by the presence of catecholamines and their metabolites, including DA, in the lacrimal fluid [23].

Thus, the secretum produced by all the glands listed above and the transudate of blood plasma penetrating through the walls of the conjunctiva capillaries constitute the fluid contained in the conjunctival cavity.

## 3. VISION DISORDERS DURING PD: CLINICAL MANIFESTATIONS AND CELLULAR AND MOLECULAR MECHANISMS

In PD, changes occur in all departments of the visual analyzer, both in the eye itself and outside the eyeball (*Table*). Thus, for example, there exists a correlation between the changes in the thickness of certain retinal sections and the structural and functional changes in the frontal and occipital cortex, as well as the development of visual-spatial cognitive impairment [68-70]. Visual impairment in PD generally manifests itself in reduced color perception, contrast sensitivity, and/or visual acuity, as well as impaired motion perception and an increased risk of hallucinations [70].

**Table:** Eye disorders detected during PD

The accessory structures of the eye	The anterior segment of the eyeball	The posterior segment of the eyeball
1. Blinking disorder [71, 75] 2. Reduced quality and changes in the lacrimal fluid composition [71, 72] 3. Blepharitis (inflammation of the edges of the eyelids) [71, 72] 4. Oculomotor disturbances (saccade deceleration, weakened convergence) [73]	1. Iris: changes in the pupillary response to light [73] 2. Ciliary body: impaired accommodation [73] 3. Cornea: thinning and reduction of the number of nerve fibers [76–78] 4. Impaired hydrodynamics: glaucoma [79] 5. Lens: cataract [80]	1. Retina: biochemical (dopamine deficiency and the presence of α-synuclein aggregates), structural (thinning of the nerve fiber layer), and bioelectric dysfunctions [68, 69, 73, 79] 2. Optic nerve atrophy [81]

In addition, the frequency of blinking and the contraction force of the smooth muscle (the orbicularis oculi muscle) may be reduced in patients with PD because of hypokinesia. These muscles surround the meibomian gland ducts and contribute to the production of meibomian gland secretum. The tear fluid outflow may also change, thus altering the evacuation of lipid secretum. Therefore, in these patients, the meibomian glands located in the eyelids can often become inflamed, which may cause blepharitis and corneal changes [71, 72].

**3.1. The retina during PD**

**Fig. 6:**
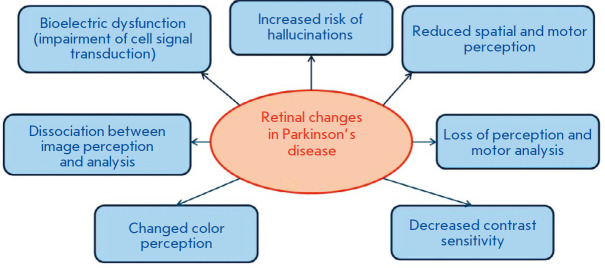
Visual impairments in PD caused by retinal changes

The retinal changes observed in PD lead to various visual impairments (*Fig. 6*) [73, 74, 75, 82].

The formation of toxic aggregates of the neuronal protein α-synuclein plays a crucial role in the pathogenesis of PD; these aggregates constitute the main component of Lewy bodies, a key marker of PD. α-Synuclein aggregation in neurons is observed in the retina of patients with PD; i.e., this protein is converted into a toxin causing neuronal degeneration, which may be one of the reasons behind the visual impairment [83]. Phosphorylated α-synuclein, which simultaneously accumulates in the brain, was detected in autopsy specimens of the retina of patients with PD [84, 85, 86, 87]. Abnormal phosphorylation of α-synuclein, as well as its intracellular aggregation, is the decisive factors and biomarkers of the pathogenesis of PD.

Significant bioelectric dysfunction (impaired signal transmission between retinal neurons) of the visual pathway in the outer retinal layers is observed in patients with PD [88, 89]. Moreover, the retina of the patients is characterized by a significantly reduced thickness of the layers of the nerve fibers and ganglion cells, as well as the inner and outer plexiform layers, while the thickness of the inner nuclear layer is increased compared to that of healthy people [90]. The inner retinal layers become thinner as BP develops. The layer of ganglion cells also changes [91]. Patients with PD were found to have scotomas (blind spots in the field of vision) even when the retinal thickness had not decreased [90, 91]. The shape of the fovea (the central fossa of the retina) is altered in patients with PD: the upper-lower tilt is flatter and the nasal-temporal tilt angle is smaller [92]. Patients with PD have an asymmetric foveal retinal thickness between the eyes [93], which correlates with asymmetric death of nigrostriatal dopaminergic neurons in the right and left hemispheres of the brain and the resulting asymmetry in the time of occurrence and in the degree of motor disorders in the extremities [94]. Optic atrophy is also detected in patients with PD [81].

**3.2. The anterior segment of the eyeball during PD**

DA, along with other neurotransmitters, affects the state of the smooth muscles of the iris which determine the pupil diameter. The parameters of the pupillary reaction to light are altered in patients with PD; it was shown in experiments on mice that exogenous dopamine causes dose-dependent pupil expansion. It is believed that this effect is caused by the conversion of dopamine to norepinephrine, which dilates the pupil by acting on the α-adrenoreceptors in the iris muscles [26]. It was shown using pupillometry (a method for recording the pupil size and the dynamics of its change) that the rate of constriction of the smooth muscles of the iris is significantly decreased, and that pupil constriction in response to light stimulus is accelerated in patients with PD [95]. Interestingly, these changes are more pronounced in patients with PD accompanied by cognitive impairment than without [96]. It is important to emphasize that hyperreaction of the pupil to parasympathomimetic and sympathomimetic effects is characteristic of PD [97], something that can probably be used to identify markers of a preclinical stage of PD and assess treatment effectiveness [98]. The non-invasive method of pupillometry may be promising in developing a prodromal (premotor) diagnosis of PD.

Significant changes in PD also occur in the cornea. In patients with PD, the corneal thickness is reduced. possibly because of the decreased frequency of blinking and the development of dry eye symptoms [76]. Misra S.L. et al. [77] reported a density of the subbasal nerve plexus (7.56 ± 2.4 mm/mm2) significantly reduced in the cornea of PD patients compared with the controls (15.91 ± 2.6 mm/mm2). The extent of the reduction in the density of subbasal nerve fibers in the cornea correlates with the degree of cognitive impairment [77, 78]. It is possible that changes in corneal innervation in PD occur before the motor function is altered [99, 100].

**3.3. The lacrimal fluid and markers of PD**

It is known that secretion of the total protein by the lacrimal gland is regulated not only by the parasympathetic and sympathetic systems, but also by the stimulation of postsynaptic D1-like receptors by dopaminergic afferents [101]. The lacrimal gland contains dopamine and dopamine receptors [102, 103]; i.e., dopamine is involved in the regulation of the amount and composition of the lacrimal fluid. It was previously shown that in the tear fluid of PD patients, the concentration of tumor necrosis factor alpha (TNF), a proinflammatory cytokine supporting progressive neurodegeneration in dopamine-containing neurons, increases significantly [62]. Furthermore, patients with PD often exhibit the dry-eye symptom [78]: tear production is reduced, and there are changes in the lacrimal film covering the cornea. On the one hand, this is caused by a reduced frequency of blinking, while, on the other hand, it is by the deterioration of the lacrimal, meibomian, and other glands. A statistically significant correlation was revealed between the severity of dry-eye signs and the stage of PD [75, 78]. Increased lacrimation is often observed concurrently with dry eyes in patients with PD. Changes in eyelid motility is a possible reason for that.

## 4. EYE DISORDERS DURING PD (GLAUCOMA AND CATARACT): CLINICAL MANIFESTATIONS AND CELLULAR AND MOLECULAR MECHANISMS

A number of studies show a relationship between the pathogenesis of PD and that of cataract (a clouding of the lens of the eye) and glaucoma (optical neuropathy due to the death of retinal ganglion cells).

**4.1. PD and glaucoma**

The risk of developing glaucoma by patients with PD increases by 30% [79]. The concentration of catecholamines, including dopamine, in aqueous humor and lacrimal fluid was found to be decreased in patients with glaucoma compared to healthy people, attesting to the fact that glaucoma causes a dysfunctioning of the dopaminergic system of the eye [79, 104]. The etiology of glaucoma, as well as that of PD, is multifactorial. It is likely that pathogenetic mechanisms are shared between the development of neurodegeneration in PD and the death of ganglionic neurons in glaucoma, such as oxidative stress and activation of microglia in nervous tissue [79].

**4.2. PD and cataract**

The cataract frequency in patients with PD is 1.48 times higher than that in patients without PD [80]. In the lens of patients with both PD and cataract, the activity of glyceraldehyde-3-phosphate dehydrogenase, which is involved in glycolysis and apoptosis induction, is lower than in patients with cataract but without PD [80, 105]. In addition, the lens of patients with PD extracted during cataract removal showed a higher content of α-synuclein than that in patients with cataract but without PD [106]. α-Synuclein accumulation and its aggregation into toxic complexes in neurons in patients with PD and in the crystalline lens of patients with cataract indicates that these pathological processes share the same mechanism.

## 5. USING THE EXPERIMENTAL MODELS OF PD TO STUDY CONCOMITANT VISION DISORDERS AND DEVELOP EARLY DIAGNOSIS

Since PD can only be diagnosed at the clinical stage of mobility impairments, changes during earlier stages can only be studied using experimental models of PD. There are various models that reproduce the degradation of the nigrostriatal system, as well as the DA deficiency in it, and induce Parkinsonism in animals. They are the genetic (knockout and transgenic) and neurotoxic models.

Modeling of PD using the proneurotoxin of dopaminergic neurons, 1-methyl-4-phenyl-1,2,3,6-tetrahydropyridine (MPTP), allows one to reproduce various stages of the disease, starting from the preclinical stage when there is no mobility impairment and ending with the late clinical, terminal stage [9, 106]. Using our models of pre-symptomatic (preclinical or prodromal) and symptomatic (analogous to clinical) stages of PD, we found that the levels of monoamines (norepinephrine, dopamine, and serotonin) in mouse eyes are reduced, an indication of the development of systemic pathological processes that extend to the eye and start before the mobility impairment appears [107]. It was also shown that systemic administration of small doses of MPTP (10 mg/kg) to mice reduces the retinal levels of dopamine metabolites (3,4-dihydroxyphenylacetic and homovanillic acids), but not dopamine. At higher doses of MPTP (30 mg/kg), the levels of both dopamine and its metabolites were significantly decreased [108]. Electroretinography (ERG) showed that after mice had receives this neurotoxin, the wave amplitude decreases, with b-wave amplitude decreasing to a greater extent than that of a-wave, which decreases only 10 days after the administration of MPTP. These changes in the ERG were normalized 50 days after the injection. In the same experiment, it was shown that 10 days after the administration of MPTP, the number of amacrine cells expressing tyrosine hydroxylase decreases by 50%, and their full recovery occurrs 50 days after the neurotoxin administration [109]. Intravenous administration of MPTP to mice showed a reversible dose-dependent decrease in tyrosine hydroxylase activity in amacrine cells [101].

In our model of the pre-symptomatic stage of Parkinsonism in mice induced by systemic administration of MPTP, we observed an abrupt increase in serotonin levels in the eyelids, while a reduced serotonin level was observed in the model of early symptomatic stage of Parkinsonism. This means that serotonin levels in the eyelids vary depending on the stage of the neurodegenerative process [108]. On the one hand, it is possible that the changes in serotonin levels affect the lacrimal fluid composition, further contributing to the onset of the dry eye syndrome and inflammation (blepharitis). On the other hand, changes in serotonin levels alter the corneal sensitivity, since serotonin is known to be involved in the sensitization of nociceptors (pain receptors) [110]. Clinical data confirm the involvement of serotonin in this process: corneal sensitivity decreases in the late stages of PD [111]. Furthermore, using this model, we revealed changes in the total protein levels in the tear fluid: protein concentration in tear fluid at the early symptomatic stage of Parkinsonism reduced abruptly: threefold (control, 3.1 ± 0.67 mg/ml; experiment, 1.1 ± 0.22 mg/ml, p < 0.04), while a downward trend was observed during the presymptomatic stage (before mobility impairments developed). This aligns with the clinical data, since a reduced level of certain proteins and changes in the protein composition of the tear fluid were also detected in patients with PD [112, 113].

We found that the norepinephrine and DA concentrations in the lacrimal fluid were elevated in patients with PD compared with the age-matched control, being more pronounced for the ipsilateral side, where motor symptoms emerge. On the contrary, the adrenaline concentration in patients’ lacrimal fluid was evenly reduced both on the ipsilateral and contralateral sides. Furthermore, the norepinephrine concentration in the lacrimal fluid was increased in patients with PD and in mice, in models of preclinical (before the motor symptoms emerged) and early clinical stages of Parkinsonism [114]. It is worth noting that changes in the levels of the protein and catecholamines in the tear fluid in experimental Parkinsonism are probably related to impaired innervation of the lacrimal gland at the stage before mobility impairments develop. Changes in the catecholamine concentration in the lacrimal fluid, and the norepinephrine concentration in particular, can be considered a marker during the early stages of PD.

Thus, experimental models of PD can be used to study the neurodegenerative processes occurring at the presymptomatic stage before mobility impairments develop, not only in the central nervous system, but also in the peripheral organs (and the eye in particular). Searching for biomarkers in the lacrimal fluid during PD is a promising strategy for developing a method for early diagnosis of this disease.

## 6. CONCLUSION

During PD, changes take place in all parts of the visual analyzer and in the auxiliary apparatus of the eye. Neurodegenerative dysfunctions occur in the retina, which shares a common embryonic origin with the central nervous system. Such dysfunctions also take place in the tissues of the anterior segment of the eye, which are involved in the regulation of intraocular pressure and accommodation, and are responsible for the pupil size. Significant changes occur on the surface of the eye: there are all signs of dry eyes and blepharitis, which most likely cause corneal thinning. The density of subbasal nerve fibers in the cornea also decreases. Modern methods of ophthalmological examination allow one to noninvasively detect the abovementioned changes and can be useful in developing a preclinical (before mobility impairments appear) diagnosis of PD. Along with ophthalmological examination, it is also possible to search for biomarkers of PD in the lacrimal fluid. The information presented in this review on the relationship between neurodegenerative processes in the brain and the eye allows one to consider the eye as a “window” that makes it possible to detect early manifestations of PD (which is very important for a successful treatment of this serious illness) and to pinpoint new pathogenetic mechanisms that underly the development of neurodegenerative processes in the eye itself.

## Abbreviations

DA: dopamine
DOPA: dihydroxyphenylalanine
DOPAC: dihydroxyphenylacetic acid
HVA: homovanillic acid
IOP: intraocular pressure
MPTP: 1-methyl-4-phenyl-1,2,3,6-tetrahydropyridine
PD: Parkinson’s disease
